# Advancing Our Understanding of Provider Behavior Change for Improved Health Outcomes

**DOI:** 10.9745/GHSP-D-23-00314

**Published:** 2023-11-30

**Authors:** Amanda Kalamar, Foyeke Oyedokun-Adebagbo, Laura Reichenbach

**Affiliations:** a Population Council, New York, NY, USA.; b U.S. Agency for International Development, Abuja, Nigeria.

## Abstract

The articles in this supplement highlight the need for strengthening the measure of provider behavior change and provide new evidence and tools for advancing our understanding of provider behavior and effective ways to ensure delivery of high-quality care that supports both clients and providers.

## INTRODUCTION

Health care providers play a critical role in the health system and are an essential component for the successful delivery and sustained uptake of health services. Understanding provider behaviors and designing interventions to strengthen their performance and contribution to the health system is a complex undertaking. Providers reflect a range of cadres with varying levels of education, training, incentives, and compensation. They engage with a diversity of patients and clients across a broad range of health services—from one-time lifesaving interventions to supporting the uptake of interventions that require sustained behavior change for addressing chronic health conditions—all of which require different provider competencies and behaviors. Providers often function in settings with unpredictable or scarce resources, including medical supplies, knowledge, and time.

Just as there is no single type of provider, there is no standard understanding of provider behavior or the best way to support provider behavior through programming. As a result, understanding the drivers of provider behavior requires examination from multiple perspectives and approaches to inform programming to best support the provider's performance. A health systems approach^1^ focuses on identifying and improving gaps within the structural environment where a provider works (i.e., availability of supplies and equipment, geographic density of health providers, clinical training and supervision, and compensation).^2^ Although structural approaches and investments that have focused mainly on clinic resources and clinical training are critical for supporting providers, they often do not take into account other drivers and influences on provider behavior, such as underlying attitudes, values, biases, and motivations. Social and behavior change (SBC) approaches can identify underlying social norms, attitudes, and other influences on provider behavior that impact their ability to deliver care and ultimately influence health outcomes.^3^ However, there has been relatively limited research and programmatic work that adopts an SBC approach that includes a focus on the multidimensional determinants of provider behavior—what motivates them and what attitudes and beliefs they should hold and why—to design and evaluate interventions to support providers and change their behaviors to improve client health outcomes.

The lack of a common definition or single understanding of PBC in the existing research and programmatic literature related to provider behavior has implications for the future of PBC investment and programmatic direction. For many, PBC is associated with quality improvement and quality of care interventions that focus on addressing structural gaps through interventions such as training and supportive supervision. However, we posit that understanding provider behavior is enhanced by adopting an SBC lens that recognizes that to be effective, PBC interventions must also address normative factors, such as underlying attitudes, values, and biases that drive a provider's behavior, in addition to the structural environment in which they work. Therefore, PBC interventions that go beyond clinical training (e.g., technical job aids) and seek to understand and positively influence provider behavior can improve the quality of services, enhance client experiences, increase demand for services, and increase uptake of commodities or adoption of healthier behaviors.^4^

This expanded the focus on and measurement of provider behavior to better inform the design and evaluation of interventions to support providers in different contexts. This is especially timely as the health system grapples with achieving major policy objectives, such as universal health coverage, and ensuring the provision of respectful care^5^^,^^6^ in an equitable manner. The current move toward service delivery interventions, such as self-care, integration, pharmacy provision, and task-shifting, requires an increased understanding of the provider's role that recognizes how providers are influenced by both structural factors (e.g., clinic resources or job aids) and nonstructural (e.g., underlying attitudes that influence how a provider might approach an interaction with a client).

## PBC: LOOKING BACK

There has been a long history of attention to quality of care in the literature—particularly in the delivery of family planning services and more recently in maternal and reproductive health services—and efforts to develop and use metrics that capture different dimensions of care.^7^ This has led to important and needed advancements in our understanding of how to improve quality of care, improve health outcomes, and ensure clients' rights.

Given the power imbalances that often exist between provider and client, much of the research, evidence, and programming has, rightfully, centered on the client to ensure that the voices of clients are heard and that the rights of clients are met.^8^^–^^10^ This continues to be needed, particularly as researchers and programmers extend the application of evidence-based quality of care to service delivery points (e.g., pharmacies and drug shops^11^) and to self-care.^12^ However, the current client experience remains influenced by the interaction with the provider and the interpersonal relationship between provider and client. A focus on the client that is too myopic risks leaving providers—as professionals and as humans shaped by experiences and norms—out of the conversation.

Quality of care frameworks that have guided research, measurement, and program implementation have helped to conceptually and visually bring together the different factors that influence the level of quality of care that is delivered and received, including client-focused components and provider-focused components. Many of the client-focused components are influenced by or dependent on the provider. For example, much of the literature on disrespect and mistreatment, particularly of pregnant, laboring, and postpartum women, has shed light on the interaction between clients and providers and the consequences that can arise from mistreatment during this interaction. There have been fewer explorations in the literature into the drivers of the provider's behavior that result in disrespect, abuse, and mistreatment. Studies that have centered on the provider have been on immediate structural influences, such as lack of infrastructure, staff shortages, and poor supervision.^13^

The Bruce-Jain framework^14^ and Donabedian model,^2^ both of which guide approaches to quality of care in family planning and reproductive health, identify different categories or groups of factors that influence quality of care. These groups of factors acknowledge the role that attributes of a service provider can play in the client-provider interaction, but these have been nearly universally measured from the perspective of the client without investigation into the drivers of or influences underlying these attributes.

Providers bring their own experiences—and potentially their own biases—to an interaction with a client. These experiences and biases can potentially overlay and impact how their clinical training is translated into practice. For example, in Rowe's health worker framework, health worker performance encompasses availability, clinical competence, responsiveness (providing client-centered care), and productivity (or efficiency).^15^ In this framework, training is a necessary component for care delivery but not sufficient. Moreover, the COVID-19 pandemic has highlighted the mental toll that being a care provider can take on individuals.^16^ This aspect of a provider is rarely addressed in current health programming.

Beyond conceptual frameworks that guide evidence generation, programs, and interventions, several large-scale efforts exist to translate these frameworks into meaningful measurement designed to monitor and evaluate the quality of care being delivered across a range of health services.

The Service Provision Assessment measures quality of care through a multimodal data collection strategy, which includes an inventory questionnaire that measures availability of services as well as structural quality indicators, a health worker interview, observation protocols, and client exit interview questionnaires.^17^ The newly revised exit interviews focus on the client's experience of care and include questions on client-provider communication and respectful treatment. The recent revision of the health worker interview captures certain human resource aspects of structural quality (i.e., professional qualifications and in-service training) as well as measures of providers' experiences of verbal and physical abuse in the workplace, work satisfaction, and management of other staff. The interview does not include questions on behavioral drivers or determinants of behavior that may influence the delivery of care and services. Similarly, Performance Monitoring for Action surveys include a sample of service delivery points for interview. However, the survey methodology does not include interviews with providers.^18^ The process aspects of quality of care, including experience of care, are measured through client exit interviews. The Quick Investigation of Quality for monitoring quality of care in family planning also uses a multimodal data collection strategy that includes a facility audit tool, an observation tool, and a client exit interview tool but does not include measurement of the provider.^19^

Important work has also been done to develop specific measures of quality of care. For example, in family planning, the Method Information Index Plus, along with a 22-item and 10-item scale, together capture critical aspects of and provide tools for monitoring and measuring process quality, but this information is collected only from the client's perspective.^7^

Without an understanding of and measurement from and about the provider, we have limited insight into effective behavior change strategies and how PBC can improve quality of care and health outcomes. Current prevalent measurement strategies have made progress, but provider behavior and its determinants are still in the shadows. Investments in PBC evidence generation, measurement, and programming can and should: (1) ensure high quality of care and improved health outcomes for clients and (2) support providers as professionals and as people and support their ability to deliver high-quality care.

## PBC: LOOKING FORWARD

Recent work, including the articles presented in this supplement, highlights encouraging innovations in PBC research, programming, and evaluation. Although there remains work to be done to refine PBC programming so that it best meets the needs of providers, there are several observations to build on to move the PBC field forward.

Adopting a PBC approach that includes a focus on influences of provider behavior, such as norms, attitudes, and biases—including how these are formed and how they can be changed—enables a more holistic view of the provider that better captures the multiple drivers of provider behavior. In the past, this has been subsumed by a focus on elements influencing provider behavior, such as clinical training and job aids. Although these interventions are necessary, they are not sufficient for addressing the totality of what influences provider behavior. The inclusion of SBC approaches that encourage an interrogation of and focus on underlying determinants of provider behavior to complement other elements allows for a more sufficient and holistic understanding that includes normative and behavioral drivers that influence or predict provider behavior.

Expanding quality of care approaches to address provider behavior and its multidimensional determinants also offers alternative strategies for improving patient outcomes beyond focusing solely on the provider. There is the potential to unintentionally blame the provider for poor patient outcomes without considering the role of the client or patient in facilitating a client-provider interaction. The experience of providers during the COVID-19 pandemic highlighted not only the global value of providers but also the vulnerabilities providers face in terms of their own mental health and the impact of burnout on their ability to perform their jobs.

PBC approaches can benefit from implementation science to address key questions related to PBC programming, such as what works, how can it work best, and how can it be replicated, scaled, and sustained. For example, determining why a PBC intervention did not work may require identifying challenges at the provider “level” that could be structural, normative, or individual. Implementation science also provides a more expansive lens through which to consider the rationale for investment in PBC approaches beyond specific health outcomes. This can contribute to the need for a clearer rationale for investment in PBC that sees the provider as more than a means to a specific health outcome.

A key to moving the PBC field forward and to answering critical questions of what works for PBC is better measurement of PBC. Although there has been progress in PBC measurement, broader questions of who is responsible for measuring and addressing PBC moving forward, what provider measurement looks like from a health systems perspective, who pays, and who invests must be addressed. Addressing these questions and moving the PBC field forward is especially important and timely given the critical role that providers play in not only improving individual-level health outcomes but also in supporting the achievement of universal health goals.

## A CALL TO ACTION: STRENGTHENING THE EVIDENCE FOR PBC APPROACHES

To continue to advance our understanding of PBC for improved health outcomes, approaches to improve the quality of care will need to commit to robust evaluation of PBC interventions to enable learning that supports policymakers to target quality improvement and invest in evidence-based behavior change programs.

Program designers should design interventions that acknowledge and address providers' behavioral determinants and ensure that these are robustly evaluated. These critical evidence-based interventions can complement other quality of care or service delivery approaches to improve health outcomes. Program implementers and researchers should consider applying both a framework that captures the system-level determinants and a behavioral theory that captures individual determinants to create a more comprehensive picture of the drivers of provider behavior.

Program designers should design interventions that acknowledge and address providers' behavioral determinants and ensure that these are robustly evaluated.

Future research will need to focus on developing and validating new measures or adapting and using existing measures to generate an evidence base that reflects the complexity and nuance of provider behavior. Moving beyond cross-sectional descriptive studies and beyond assessing skills and training-based approaches, measurements of core concepts of provider behavior, such as provider attitudes and provider bias, are needed to concretely assess and address provider performance. Evaluations of PBC interventions should use a multimodal data collection approach to collect both provider-level and client-level outcomes to help elucidate how changing provider behavior is linked with improved client outcomes for family planning.

In support of our call to action, the articles in this supplement highlight the need for strengthening the measure of PBC and provide new evidence and tools for advancing our understanding of provider behavior and effective ways to ensure delivery of high-quality care that supports both clients and providers. The articles consider how PBC is defined and understood, interrogate existing measurement, propose new measurement tools, and share needed evidence from evaluations of PBC programming approaches with recommendations for the future.

Hancock et al.^20^ make a case for viewing providers and provider behavior through a holistic lens that considers the full complement of provider types and the full set of factors that may influence their behavior and, in turn, provider-client interaction.

To address global measurement gaps in PBC, Dougherty et al.^21^ investigate the current state of PBC measurement to identify gaps and opportunities, and Silva et al.^22^ delve specifically into the measurement of provider attitudes that can influence their behavior. Dougherty et al. identify an important opportunity to strengthen the role of behavior change theory in the measurement of interventions designed to address provider behavior and/or improve provider/client interactions. Silva et al. put forth a measurement tool developed to measure critical constructs of provider behavior in line with established behavioral theories.

To help shed light on specific influences on provider behavior, Sripad et al.^23^ use a gender and power lens to better understand providers' experiences and behavior, drawing on data from Kenya, Malawi, Madagascar, and Togo. Burnett-Zieman et al.^24^ investigate the critically important issue of provider mental health in Malawi, finding that addressing burnout is critical for improving respectful care. The influences raised in these articles help to contextualize provider behavior and serve as an important reminder of the human nature of health care.

And finally, Smith et al.^25^ and Warren et al.^26^ share experiences and lessons from implementing PBC interventions. Warren et al. demonstrate the successful application of a nurturing, integrative, and responsive approach in the care of hospitalized newborns and young children in Kenya. Smith et al. highlight the successes that can be achieved when a behavioral science lens is used to strengthen interventions and implementation.

Collectively, the articles in this supplement guide the way forward to strengthened measurement and implementation of PBC interventions for improving health outcomes.

## Notes

**Peer Reviewed**
